# Co-cultivation and transcriptome sequencing of two co-existing fish pathogens *Moritella viscosa* and *Aliivibrio wodanis*

**DOI:** 10.1186/s12864-015-1669-z

**Published:** 2015-06-10

**Authors:** Erik Hjerde, Christian Karlsen, Henning Sørum, Julian Parkhill, Nils Peder Willassen, Nicholas R. Thomson

**Affiliations:** Department of Chemistry, Faculty of Science and Technology, University of Tromsø, N-9037 Tromsø, Norway; Section of Microbiology, Immunology and Parasitology, Department of Food Safety and Infection Biology, Faculty of Veterinary Medicine and Biosciences, Norwegian University of Life Sciences, Ullevålsveien 72, Oslo, Norway; The Pathogen Sequencing Unit, The Wellcome Trust Sanger Institute, Wellcome Trust Genome Campus, Hinxton, Cambridge, CB10 1SA UK; The Norwegian Structural Biology Centre, University of Tromsø, N-9037, Tromsø, Norway

**Keywords:** *Aliivibrio*, *Moritella*, Complete genome, RNA sequencing, Co-culture, Co-infection, Bacteriocin, Winter-ulcer

## Abstract

**Background:**

*Aliivibrio wodanis* and *Moritella viscosa* have often been isolated concurrently from fish with winter-ulcer disease. Little is known about the interaction between the two bacterial species and how the presence of one bacterial species affects the behaviour of the other.

**Results:**

The impact on bacterial growth in co-culture was investigated *in vitro*, and the presence of *A. wodanis* has an inhibitorial effect on *M. viscosa*. Further, we have sequenced the complete genomes of these two marine Gram-negative species, and have performed transcriptome analysis of the bacterial gene expression levels from *in vivo* samples. Using bacterial implants in the fish abdomen, we demonstrate that the presence of *A. wodanis* is altering the gene expression levels of *M. viscosa* compared to when the bacteria are implanted separately.

**Conclusions:**

From expression profiling of the transcriptomes, it is evident that the presence of *A. wodanis* is altering the global gene expression of *M. viscosa*. Co-cultivation studies showed that *A. wodanis* is impeding the growth of *M. viscosa*, and that the inhibitorial effect is not contact-dependent.

**Electronic supplementary material:**

The online version of this article (doi:10.1186/s12864-015-1669-z) contains supplementary material, which is available to authorized users.

## Background

Winter-ulcer disease affects reared salmonids when seawater temperatures drop below 8 °C [1]. *Moritella viscosa* is the aetiological agent of the disease. The significance of *Aliivibrio wodanis*, which is often co-isolated with *M. viscosa* or as the only isolate from diseased fish is uncertain [2]. Both *M. viscosa* and *A. wodanis* are Gram-negative gammaproteobacteria. The *Moritella* genus is a member of the Moritellaceae family, and consists mainly of psychrophilic and barophilic species isolated from deep-sea or marine sediments. *M. viscosa* is the only currently known pathogenic member of the *Moritella* genus. *A. wodanis* belongs to the *Aliivibrio* genus of the Vibrionaceae family. Several species from this family are pathogens causing diseases in different marine animals. *A. wodanis* will be the second *Aliivibrio* species with a complete genome available.

Both *M. viscosa* and *A. wodanis* are cytotoxic to fish cells [3]. *M. viscosa* 06/09/139 and *A. wodanis* 06/09/139, which are genome sequenced in this study, are also both separately producing clinically disease symptoms in Atlantic salmon *Salmo salar* in bath challenge and are able to co-infect Atlantic salmon in a bath co-infection model [3]. The role of this bacterial interplay in the pathogenesis of winter-ulcer is not known, but *A. wodanis* colonisation of fish surfaces is hypothesized to influence the progression of a *M. viscosa* infection.

Since the two bacteria often co-exist in ulcers and internal organs, it is likely that they interact with each other. Interspecies interactions have been extensively explored within oral microbial communities (reviewed in [4] and [5]). Similarly, interactions in multimicrobial communities in fish hosts might be beneficial through synergistic effects (e.g., utilization of metabolic products, biofilm formation) communication (e.g., quorum sensing), or competition (e.g., for nutritional resources).

A coordinated behaviour involving intercellular communication between two species could be regulated via the production and response to signal molecules [6]. This cell-density dependent regulation termed quorum sensing (QS), is likely to provide a selective advantage for both bacterial populations by allowing them to alter their morphology and physiology quickly to adapt to environmental changes. Many Gram-negative bacteria use N-acyl homoserine lactones (AHLs) as QS signal molecules, and AHLs from one species can regulate the behaviour of another species. For example *Escherichia coli* are able detect and regulate gene transcription in response to AHLs without having the ability to produce the molecule itself [7]. In addition to AHLs, many Gram-negative bacteria produce and recognize an autoinducer called AI-2. The widespread existence of this signal molecule indicate that bacteria can communicate across species boundaries using AI-2 [8].

On the other hand, within a mixed bacterial community the bacteria may compete with their neighbours for space and resources. There are several mechanisms by which bacterial species can dominate and outcompete other organisms, but nutritional resources seems to be a focal point of microbial competition (reviewed in [9]). The ability to grow faster or to acquire nutrition more efficiently by one organism comes at the expense of another. For example, an important mechanism for the uptake of iron is mediated through siderophores. Siderophores are iron-scavenging molecules that are produced and secreted from the bacterial cells, and actively transported back into the cells when they have sequestered iron from the surrounding environment. The structure of over hundred terrestrial siderophores with different affinity to bind iron has been described. On the other hand, the structure of only a few marine siderophores is known, the majority belong to the family of amphiphiles. Many amphiphilic siderophores are anchored to the cell through one or more fatty acid appendages, which is an adaptation to the low abundance of iron in seawater [10]. It may also be advantageous for bacteria living in different environments, e.g., the seawater and inside a host, to be able to utilize different siderophores. Further, one bacterial species could be outcompeted by the presence of another that produces a high-affinity siderophore [11].

Bacteria can also inhibit growth and kill competitors by secreting antimicrobial compounds toxic to other bacteria. Bacteriocins are proteinaceous molecules that inhibit growth of similar or closely related bacteria species through lethal disruption of membrane potential and integrity [12]. Some bacteriocins like vibriocins produced by several members of the Vibrionaceae family have toxic effects on more distantly related bacteria [13]. In Gram-negative bacteria a conserved leader sequence termed the GG-motif [M(R/K)ELX3E(I/L)*X*2(I/V)XG(G/A)] has been determined in the N-terminal part of bacteriocins [14]. This leader sequence is cleaved off by a transport peptidase which is often found together in the bacteriocin locus along with a HlyD family protein involved in the transport across the membrane [15]. Self-protection against the bacteriocin are thought to be encoded by abortive infection (*abi*) genes which also can be found in the bacteriocin locus [16].

To investigate if there exist any synergistic effects between *M. viscosa* and *A. wodanis* during the course of an infection, we first performed *in vitro* mono- and co-culture growth studies in different media. We then applied Illumina technology to perform high-throughput sequencing of single-stranded cDNA from the transcriptomes of *M. viscosa* and *A. wodanis.* The RNA samples were collected from *in vivo* challenge studies on fish where the bacteria were grown in separate semi-permeable tubings in the abdomen, either individually or together in the same fish. Our results show that the gene expression levels and the growth of both bacteria alters from single culture and single infection to co-culture and co-infections, and that the changes are most profound for *M. viscosa.*

## Results

### General features of the genomes

The general features of the *M. viscosa* 06/09/139 and *A. wodanis* 06/09/139 genomes are summarized in Table 1 and graphically presented in Additional file 1. The genome of *M. viscosa* has one chromosome (5.1 Mb) and two small cryptic plasmids named pMVIS41 (4.1 kb) and pMVIS39 (3.9 kb). The overall GC content is 39.4 %. The genome of *A. wodanis* has two chromosomes (3.0 Mb and 1.5 Mb), and four plasmids named pAWOD920 (92 kb), pAWOD150 (15 kb), pAWOD72 (7.2 kb) and pAWOD19 (2.1 kb). The overall GC content is 38.5 %.

**Table 1:** General genome features of M. viscosa 06/09/139 and A. wodanis 06/09/139

	Genome size (bp)	G + C (%)	# of CDSs	Coding (%)	# of rRNA operons	# of tRNAs	# of sRNAs	CDSs with TMHs	CDSs with signal peptides	Putative uncharacterized proteins	Pseudo/partial CDSs	IS elements
*M. viscosa* 06/09/139	5093989	39.43	4445	83.7	11	131	261	1076	728	634	163	49
Chromosome	5086074	39.44	4436	83.8	11	131	261	1074	727	630	163	49
Plasmid (pMVIS41)	4059	31.21	5	63.0	0	0	0	0	0	1	0	0
Plasmid (pMVIS39)	3856	36.36	4	35.9	0	0	0	2	1	3	0	0
*A. wodanis* 06/09/139	4635126	38.48	4079	86.9	7	88	95	1048	1185	546	104	27
Chromosome I	3003353	38.88	2640	87.3	6	69	69	658	519	280	48	16
Chromosome II	1515310	37.72	1313	87.0	1	19	19	369	650	222	46	4
Plasmid (pAWOD920)	91951	38.33	96	80.4	0	0	7	20	16	26	7	7
Plasmid (pAWOD150)	15266	36.22	18	72.0	0	0	0	1	0	12	1	0
Plasmid (pAWOD72)	7177	34.86	9	69.7	0	0	0	0	0	5	0	0
Plasmid (pAWOD19)	2069	37.46	3	78.7	0	0	0	0	0	1	0	0

### Growth and co-cultivation

**Fig. 1:**
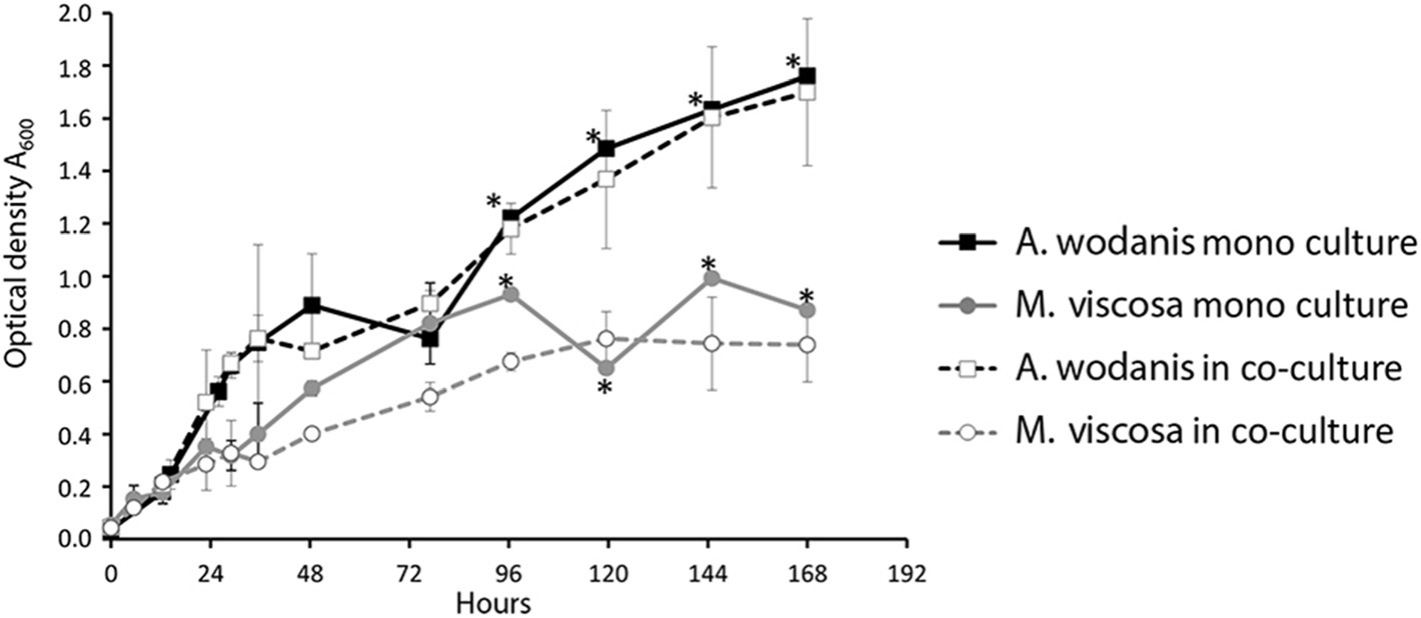
*Aliivibrio wodanis* and *Moritella viscosa in vitro* growth curves. Mono-cultures of *A. wodanis* grows faster than *M. viscosa*, and *A. wodanis* inhibits *M. viscosa* growth when cultured in co-culture. The bacteria were grown in tubings; either as a mono-culture or in two separate tubings within the same culture system (co-cultivated). The growth was measured in optical density (A_600_) and is presented as the average ± standard deviation of three parallell experiments. The symbol * present at mono-culture sample time points from 96 to 168 h indicate A_600_ values obtained from only a single culture experiment representative

**Fig. 2:**
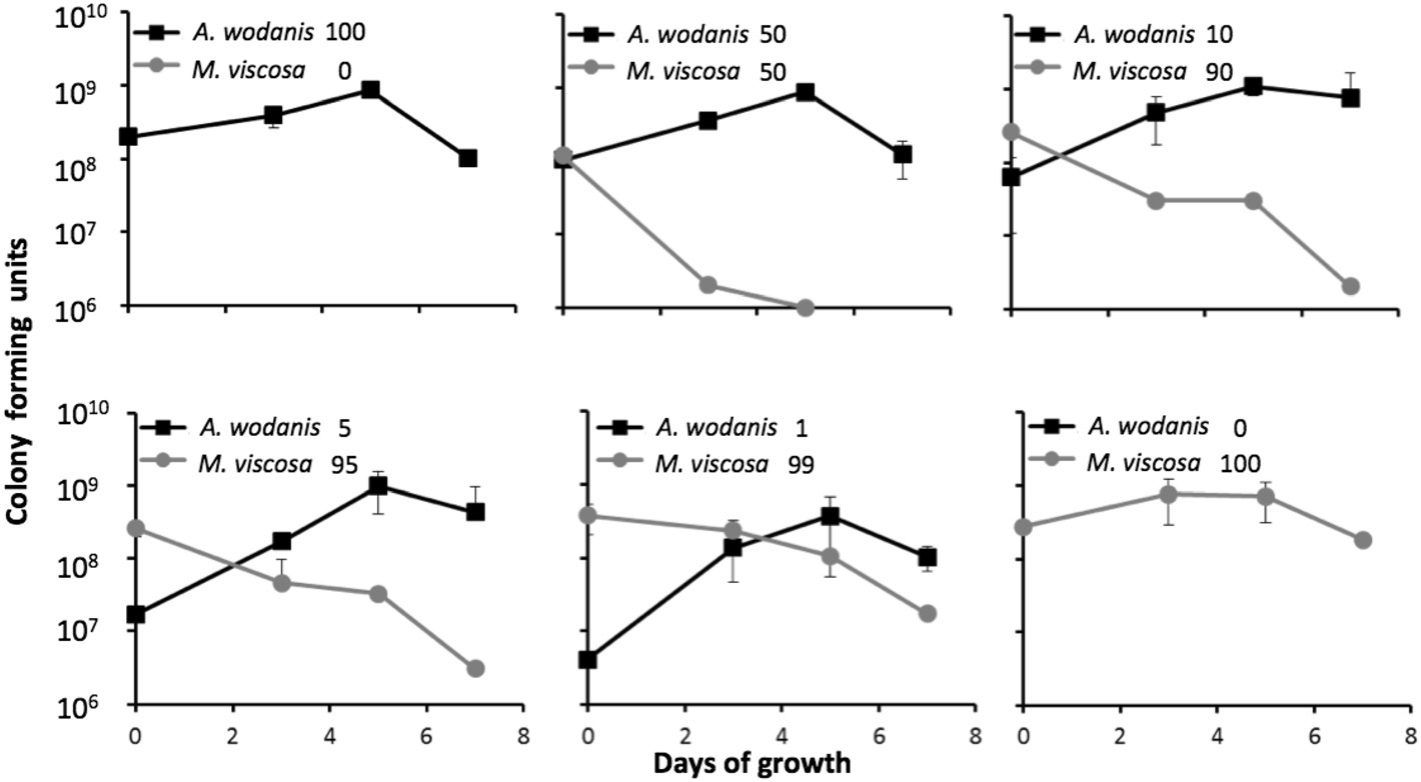
Bacterial growth in mono- and co-culture on BA plates. The growth was measured by colony growth distribution from initial *A. wodanis*: *M. viscosa* culture mixtures with the following proportions; 100:0, 50:50, 10:90, 5:95, 1:99 and 0:100. The growth development within the colonies are estimates from two parallell experiments presented as the average ± standard deviation of the colony forming units (cfu) determined. Samples were collected at initiation and after 3, 5 and 7 days

The *in vitro* growth of the two bacteria was determined in both mono- and co-cultures. Mono-cultures of *A. wodanis* are growing faster than *M. viscosa* under experimental tube-settings used in this study (Fig. 1). Typical growth conditions utilizing broth in shaked culture flasks (Additional file 2) confirmed that *A. wodanis* grows faster than *M. viscosa* at 7 °C. An increase of NaCl in the growth medium from 1.0 % to 3.5 % produced a distinct change in the growth kinetic of *M. viscosa*. A higher growth rate was observed for *M. viscosa* at 3.5 % NaCl. *M. viscosa* growth is inhibited when grown in co-cultivation with *A. wodanis. M. viscosa* kept separate from *A. wodanis* by semipermeable tubings in the same cultivation system produced a lower growth rate compared to when *M. viscosa* where cultivated in semipermeable tubings alone (Fig. 1). Co-cultivation exerted less influence on *A. wodanis*. Growth development in a colony co-culture showed that the growth of *M. viscosa* is clearly inhibited in co-culture with *A. wodanis* (Fig. 2). The inhibitorial effect is observed in all relative initial inocula-ratios tested between *M. viscosa* and *A. wodanis* from 50:50 down to 99:1. Growth inhibition or survival of *M. viscosa* corresponds to the initial *M. viscosa* : *A. wodanis* proportion i.e., *M. viscosa* is more strongly affected in high *A. wodanis*-proportion co-culture colonies. In the same co-culture colonies the growth of *A. wodanis* increases until day 5 before a decrease in cfu’s are observed, similar to the *A. wodanis* colony reference.

### Transcriptomics

At the start of the experiment three biological replicates (three salmons) were used for each sample condition. Unfortunately the fish mortality rate was high and we only managed to recover and isolate bacterial RNA from one biological replicate for each sample condition. The gene expression levels of both *M. viscosa* and *A. wodanis* are affected during co-infections relative to single infections, although the changes are most profound for *M. viscosa*. In co-infections versus single infections, 115 *M. viscosa* genes had two times lower expression value and p-value lower than 0.05 (Additional file 3a). A striking large number of these genes are involved in protein synthesis; 62 tRNAs, 11 rRNAs and three ribosomal proteins. The remaining genes encode various functions, some related to transcription, replication and stress adaptation; two DNA binding proteins, one sigma-54 modulation protein, one cell division protein, a universal stress protein, and a phage-shock protein. In total there were 49 genes with increased expression in co- versus single infections. Most of these genes have unknown functions, including the genes on plasmid pMVIS41 that seems to be up-regulated 2–4 fold when *A. wodanis* is present (Additional file 3b).

In co-infections, 87 *A. wodanis* genes had two times lower expression (p-value < 0.05) relative to single infections. Similar to *M. viscosa*, many of these genes have functions related to protein synthesis and stress adaptation (Additional file 3c). In addition, 18 genes are probably exported or are membrane proteins. At the same time, 103 *A. wodanis* genes were two times up-regulated in co-infections versus single infections. Over half of these genes (56 genes) are located on the four plasmids, most with unknown functions. Among up-regulated chromosomal genes there are several with functions related to transcription, including the RNA polymerase sigma 70 factor (Additional file 3d).

### Species-specific genes–nutritional advantage

All enzymes and the metabolic pathways in both genomes were assessed using Priam. The two complete sets of enzymes were compared and mapped to KEGG using KEGG Mapper. In total *M. viscosa* and *A. wodanis* shared 873 enzymes, *A. wodanis* had 119 unique enzymes not shared with *M. viscosa*, while *M. viscosa* had 214 unique enzymes.

*M. viscosa* carry a potential siderophore biosynthesis operon of four genes (MVIS_0633-0636), including a gene with 75 % similarity to *Pseudomonas aeruginosa pvdA*. This gene encodes L-ornithine N5-oxygenase, a key enzyme in the biosynthesis of the siderophore pyoverdin [17]. Siderophore biosynthesis, secretion and uptake in *A. wodanis* could be encoded by a locus (AWOD_I_1553-1563) that is highly similar and synthenic (Additional file 4) to the vibrioferrin locus of *V. parahaemolyticus* [18]. In addition *A. wodanis* carry a second siderophore uptake system (AWOD_II_0927-0923). The genes are over 90 % similar at amino acids level to ferric anguibactin/vulnibactin uptake systems of several *Moritella* species, including *M. marina* [19].

### Inhibitorial factors

**Fig. 3:**
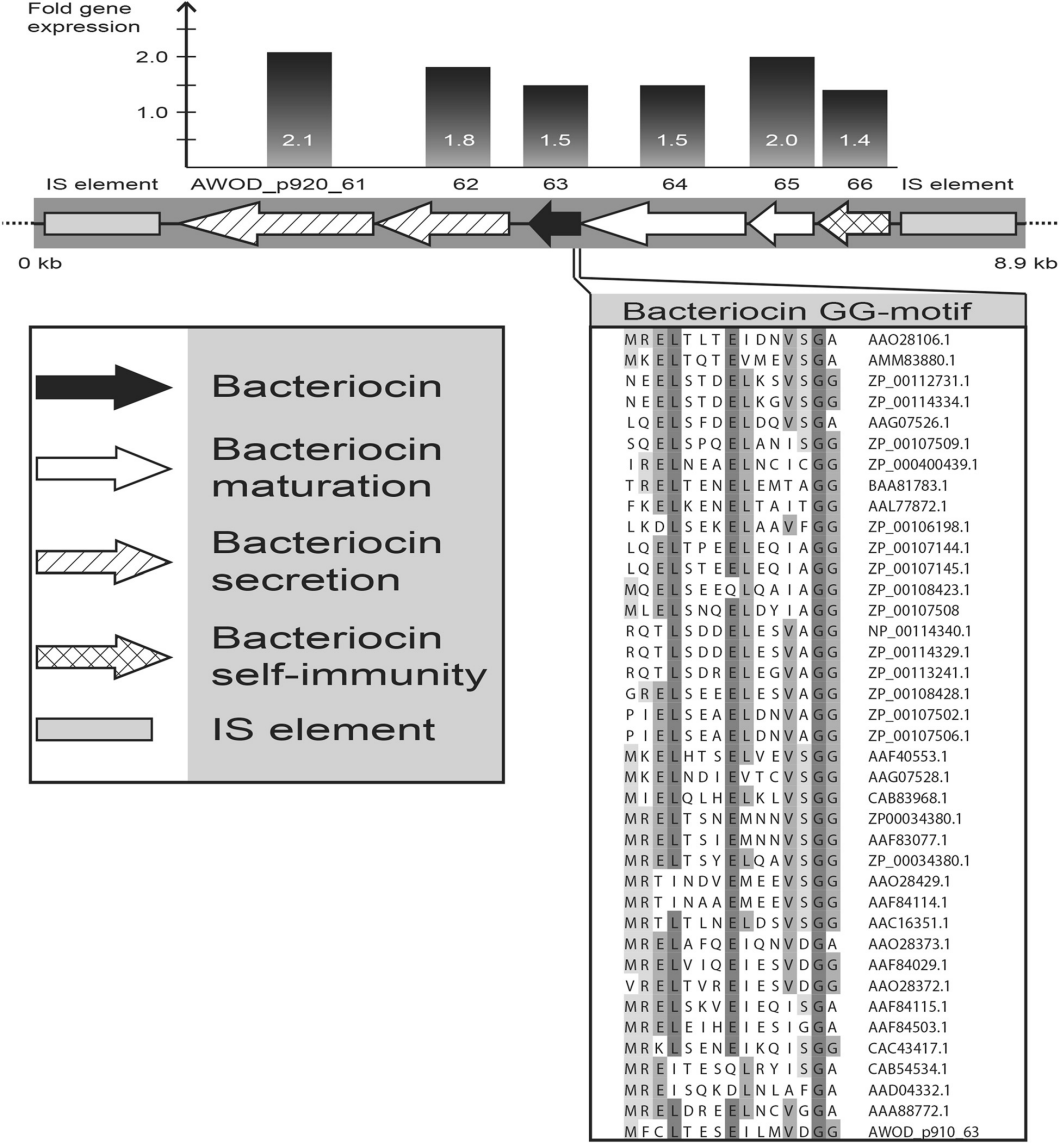
Bacteriocin gene cluster identified on *A. wodanis* pAWOD920. The differential gene expression in co-infection versus single infection are indicated above each gene. Below the bacteriocin encoding gene (black arrow) is the conserved leader peptide sequence (GG-motif) aligned against homologous bacteriocins. GeneBank accession numbers are indicated

The largest *A. wodanis* plasmid pAWOD920 carry six genes that could be involved in the production and secretion of bacteriocin (Fig. 3). The amino acid sequence of the putative bacteriocin (AWOD_p920_63) shows no similarity to any proteins in GenBank, but has the conserved GG-motif (Fig. 3) necessary for secretion [14]. The two subsequent genes AWOD_p920_61 and AWOD_p920_62 encode bacteriocin secretion proteins possible homologous to *Klebsiella pneumoniae* RYC492 *mceG* and *mceH*. Their translated products are 62 % and 46 % similar, respectively. AWOD_p920_61 encodes a transport peptidase with the conserved C/H-motifs [(QX4[D/E]C[G/A]XAXLX2[I/V]X4GX4[I/L]X2LR) and (H[Y/F][Y/V]V[L/V]X9[I/L/V]XDP)] and is probably responsible for cleaving off the bacteriocin leader sequence [20]. AWOD_p920_62 encode a HlyD family membrane fusion protein, which is thought to play a role in transport across the outer membrane. AWOD_p920_64 and AWOD_p920_65 may be homologs of *K. pneumoniae* RYC492 *mceI* and *mceJ*, both with similarities of 46 % at the amino acid level, *mceI* and *mceJ* encode proteins involved in bacteriocin maturation [21]. AWOD_p920_66 encodes a CAAX protease belonging to the Abi family (PF02517) and may be related to self-immunity.

**Fig. 4:**
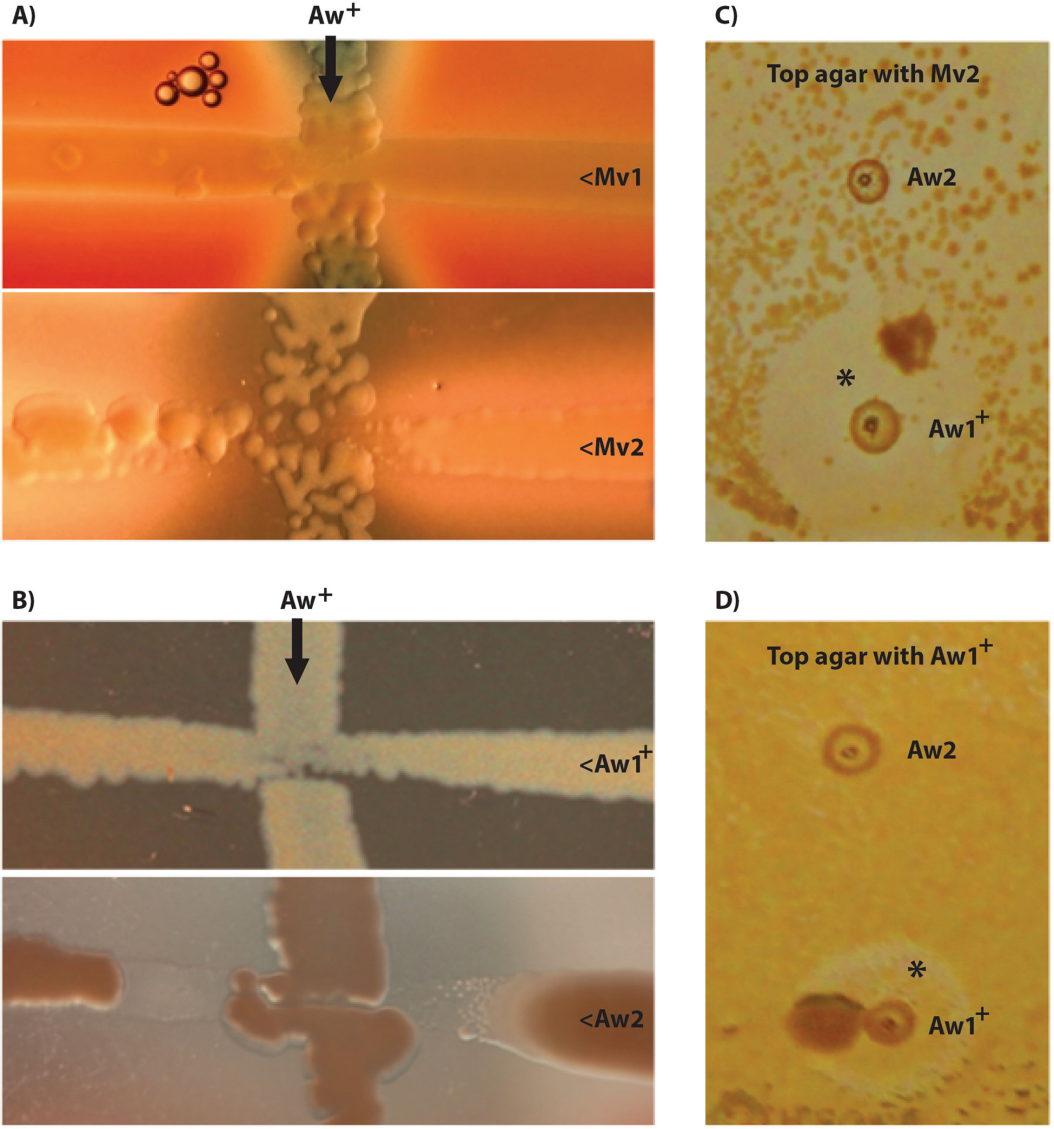
Growth and inhibition zones from bacterial cross streaks and on soft agar overlays. Representative *A. wodanis* strains positive for the bacteriocin locus (Aw^+^) were vertical streaked onto blood agar plates: A) *A. wodanis* 88/09/441, B) *A. wodanis* 06/09/139. Prependicular and in horizontal streaks: Mv1, *M. viscosa* 3632; Mv2, *M. viscosa* 06/09/139; Aw1^+^ (positive for the bacteriocin locus), *A. wodanis* 90/09/325; Aw2, *A. wodanis* 02/09/569. Reduced growth in the vicinity of Aw^+^ are illustrated for both *M. viscosa* (**a**) with Mv2 and for *A. wodanis* (**b**) with Aw2 where a clearly defined inhibition zone is observed. In the agar overlay method (**c**) and (D Aw1^+^ and Aw2 were first spotted onto the plates and grown to smal colonies before the agar overlay was added and bacterial suspensions of Mv2 (C) and Aw1^+^ (**d**) were plated on top. Colonies (Aw1^+^) surrounded by inhibition zones (marked with *) in the on-top bacterial lawn indicate bacteriocin activity

Prevalence of four bacteriocin-locus genes were assessed by PCR on a collection of *A. wodanis* isolates, which allowed the discrimination between bacteriocin-locus positive and negative isolates (Additional file 5). However, the three isolates that were found negative for the bacteriocin-locus produced PCR products from primers targeting bacteriocin secretion genes, although the product from one of the two genes were ~300 bp larger than expected (Additional file 5). Four bacteriocin carrying isolates, including *A. wodanis* 06/09/139, were screened for bacteriocin production using four *M. viscosa* isolates as indicator organisms. In parallel, the same screening was performed on *A. wodanis* that did and that did not carry the genotypes (Additional file 5). Both on the blood agar (BA) streak and in the soft agar overlay assay, inhibitorial activity against several *M. viscosa* isolates were observed (Fig. 4 and Additional file 6). *M. viscosa* isolates also appear to be differently affected by *A. wodanis*, which is illustrated in Fig. 4a where *M. viscosa* isolates responds differently in growth to one *A. wodanis* strain. Growth inhibition was reproduced in the overlay assay (Fig. 4c) where the effect was more profound. The inhibitorial activity was also tested against *A. wodanis* isolates, and cross streaking revealed that the antimicrobial activity was strongest between *A. wodanis* isolates compared to the more distantly related *M. viscosa* isolates (Fig. 4ab, Additional file 6). *A. wodanis* isolates with the bacteriocin operon did not exert any strong inhibitorial effect on itself compared to *A. wodanis* without the bacteriocin operon when cross streaked simultaneously (Fig. 4b). However, a colony inhibited growth of the same strain when it was plated on the overlay agar (Fig. 4d) indicating that the older colony could inhibit growth on non-adapted cells of the same strain.

## Discussion

When two different bacterial species are placed together in the same environment, there are at least three possible outcomes from this close encounter: The presence of a second bacterial species is not affecting the behaviour of the first; the two bacteria team up and aid each other in invading the nutrient source (for instance a host); or the two bacteria compete with each other for the same pool of resources.

The *in vitro* mono- and co-cultivation demonstrated that growth of the two bacteria is negatively affected by the presence of the other. Most profound was the impact *A. wodanis* had on *M. viscosa* growth. It is therefore likely that the relationship between the two species is of a competitive nature and that *A. wodanis* is better adapted to propagate when the two species occupies the same environment. This growth inhibition is not contact-dependent since the effect is similar when the two bacterial species are present in the same tubings as when they are in separate tubings.

Expression profiling of the transcriptome allows us to study the dynamic picture of regulatory and functional processes during adaptation to environmental changes. Gene expression profiles were measured when the bacteria were implanted together in the same fish and when they were implanted separately in different fishes. The effects *M. viscosa* and *A. wodanis* have on altering the global expression pattern in the presence of each other are discussed together with growth data from the mono- and co-culture growth experiments within the following sections. However, in the absence of biological replicates only careful interpretation of the general expression profiles are made.

### Growth and co-cultivation

Multiple copies of ribosomal RNAs (rRNAs) are assumed to reflect the ability for bacteria to grow fast. Moreover, there is a correlation between the copy number of rRNA genes and the rate at which bacteria respond to resource availability [22]. Short replication time is an evolutionary adaptation advantageous in a competitive environment with limited access to nutrients. Although the *M. viscosa* genome is only 9 % larger than *A. wodanis*, it contains almost twice the number of ribosomal RNA operons (11 versus 6 copies). From this one could assume that *M. viscosa* would grow faster than *A. wodanis*, however, under the growth conditions used in this study the opposite is the case (Fig. 1, Additional file 2).

In co-culture on blood agar, *A. wodanis* quickly becomes the dominant bacteria and outcompete *M. viscosa*, even when the initial ratio was 1:99 (Fig. 2). A similar impediment of *M. viscosa* growth is observed when they are co-cultured in LB medium (Fig. 1). In contrast, *A. wodanis* appear to be less affected when in co-culture (Figs. 1 and 2). The transcriptomic effect is strongest when the two bacteria are together in the same tubings, but the inhibition does not seem to be contact-dependent as *M. viscosa* growth also is impeded when co-cultivated in separate tubings.

The general trends from the *in vivo* gene expression profiling of the transcriptomes was that genes with similar functions are down-regulated in both bacteria during co-infection relative to when they were implanted alone in the fish. We were not able to follow the growth rate of the bacteria in the implanted tubings and thus establish if any reduction in growth or if difference in gene expression is a result of nutritional deprivation of the environment. However, culture densities from growth in tubings (Fig. 1) and estimated cfu’s on agar plates (Fig. 2) are still increasing for both *A. wodanis* and *M. viscosa* after four days of cultivation, which is the time point of when the implanted tubings were extracted from the fish. The semipermeable membrane is also likely to diffuse factors (nutritional, immune components, etc.) that could influence growth within the abdominal cavity of Atlantic salmon. However, to determine effects of such interactions with the host is not within the scope of this study. In the presence of a putative competitive bacterial species, the RNA expression profiles suggest a negative gene regulation effect, particularly on genes involved in protein synthesis. Additionally, the expression of several genes related to stress adaptation, replication and transcription are also repressed in co-infections. The negative regulation of the protein synthesis is most profound for *M. viscosa*, where all 11 ribosomal RNAs and 106 out of the total 133 tRNAs (albeit only 53 genes with p-value < 0.05) are more than two fold down-regulated in the presence of *A. wodanis*. In addition, the expression of three ribosomal proteins is two fold lower in co-infections. The abundance of RNA polymerases and ribosomes are important growth rate-dependent parameters, and the rate-limiting step in ribosome synthesis is the synthesis of rRNA. When the nutritional availability is low, the synthesis of ribosomes decreases, which in turn slows protein production and lowers the growth rate in order to conserve the cell’s energy (reviewed in [23]). It remains to be elucidated, but the reduced expression of *M. viscosa* genes involved in protein synthesis could be a result of *A. wodanis* being a more efficient in utilizing the nutrition available within the host environment, thereby preventing *M. viscosa* to meet its nutritional needs.

### Species-specific genes–nutritional advantage

The abundance of iron in the marine environment is scarce. Also for pathogenic marine bacteria there is a constant battle for iron between the invading bacteria and the host. The uptake of iron is efficiently mediated through siderophores produced by the bacteria that scavenge extracellular iron [24]. The siderophore–iron complex is very beneficial when it is transported back into the cell, but it also comes at a cost; siderophore biosynthesis requires energy and the proper assembly of the components that constitutes the functional groups of the molecule.

Björnsdottir and colleagues showed that *M. viscosa* produces siderophores, but the method they used is universal and do not discriminate between various types of siderophores [25]. From the genome sequence alone we were not able to identify the genes responsible for the biosynthesis of these siderophores. However, the chromosome carries a four-gene operon similar to the siderophore pyoverdin biosynthesis genes, which is linked to biofilm formation in *Pseudomonas aeruginosa* [17]. The largest *A. wodanis* chromosome carries genes homologous to those responsible for vibrioferrin biosynthesis and uptake in *V. parahaemolyticus* [18]. *A. wodanis* also encodes a second siderophore uptake system, with similarities to anguibactin/vulnibactin acquisition systems found in other *Moritella* species [19], but lack the corresponding siderophore biosynthesis genes. Unlike the majority of marine siderophores, neither vibrioferrin nor anguibactin/vulnibactin are amphiphilic siderophores [10], and are secreted from the cell to acquire iron. During an infection, *A. wodanis* and *M. viscosa* have to compete against the host and each other to sequester iron. Siderophore-mediated interspecies competition has been shown for other bacteria (reviewed in [9]). Since the two bacterial species seem to exhibit different types of siderophores, it is possible that *A. wodanis* produce siderophores with higher affinity for iron than *M. viscosa*. It is also possible that *A. wodanis* have retained the second siderophore uptake system as an agent to “steal” exogenous siderophores, hence putting the burden of producing siderophore on other species like *M. viscosa*. A similar strategy has been demonstrated with the ferric aerobactin receptor (IutA) mediated uptake of aerobactin by *V. parahaemolyticus* [26]*.*

### Inhibitors

Bacteria are able to outcompete other bacteria by secreting effector molecules. The impediment of *M. viscosa* growth in the presence of *A. wodanis* could be a result of inhibiting bacteriocin activity mediated by *A. wodanis*. Bacteriocin production has been reported for many members of the Vibrionaceae family ([20], [27], [28] and [29]), but the genotypes have to our knowledge not been determined. The *A. wodanis* plasmid pAWOD920 carries a locus of six genes (AWOD_p920_0066-0061) with all the functions necessary for the biosynthesis and export of bacteriocin (Fig. 3).

The majority of the genes located on *A. wodanis* plasmids have increased expression in co-infection relative to single infection. All the six genes in the bacteriocin locus are up-regulated 1.4–2.1 fold when *A. wodanis* was implanted together with *M. viscosa* in the same fish relative to when implanted separately in different fishes (Fig. 3). Whether this is a direct effect of gene regulation or the increase in plasmid copy numbers is not clear, but the translational outcome would be similar, and could give *A. wodanis* a competitive advantage over *M. viscosa*.

*A. wodanis* isolates had a strong inhibitorial effect on each other. From the cross streaking assay *A. wodanis* isolates carrying the bacteriocin-locus inhibited growth more strongly in *A. wodanis* isolates not predicted to carry this locus (Additional file 5). However, *A. wodanis* without this locus also inhibited growth on bacteriocin-locus carrying *A. wodanis* isolates. The PCR screen predicting the presence of the bacteriocin locus in *A. wodanis* isolates produced amplicons for genes predicted to secrete the bacteriocin precursor in all *A. wodanis* isolates tested (Additional file 5). A second bacteriocin system could be present in these isolates. The overlay assay displays the antimicrobial potential between both *A. wodanis* types including self-inhibition. This observation is presumably a consequence of that the older colony underneath the overlay plated bacteria is already producing the bacteriocin and the immunity protein, which confer resistance. The overlay-plated bacteria are not bacteriocin producing cells and have no immunity to the underlying bacteriocin producing cells. The inhibitorial effect is likely mediated through a diffusible agent. Both the growth inhibition zones around the colonies on the soft agar overlay and the reduced growth in the vicinity prior to the intersection point where the bacteria make physical contact on the BA plates (Fig. 4 and Additional file 6), demonstrates that the growth inhibition is not contact dependent.

Bacteriocin normally inhibits closely related bacterial species, although bacteriocins produced by other *Vibrionaceaes* have toxic effects on distantly related bacteria [12]. In accordance with this, the antimicrobial effect is stronger against other *A. wodanis* isolates than against *M. viscosa* (Fig. 4). Our results demonstrates that *M. viscosa* is susceptible to secreted agent(s) produced by *A. wodanis*, and that a likely candidate may be produced by the plasmid encoded bacteriocin locus.

### Quorum sensing

When exploring the relationship between two bacteria, it is natural to consider the possibility that the interaction could be controlled through signal molecules of QS systems. QS-communication between different bacteria imply that the signal from one species must be detected and incorporated by the second species. Bacteria that have an incomplete QS circuit may retain the ability to detect and regulate gene transcription in response to AHL without having the ability to produce the molecule itself [30].

*A. wodanis* 06/09/139 is producing the AHL N-3-hydroxy-decanoyl-L-homoserine lactone (3-OH-C10-HSL) [31], while *M. viscosa* does not produce AHLs [32]. To our knowledge, production of AI-2 has not been assessed for these bacteria. In *A. wodanis*, the AinS/AinR (AWOD_I_1040/1039) system probably confers to the 3-OH-C10-HSL production. In addition, the genome also harbours a LuxS-LuxPQ (AWOD_I_0523–AWOD_I_0676/0677) system. This system utilises AI-2 synthesised by LuxS. Both systems converge and mediate their response through a cascade of phosphorylation reactions of LuxU (AWOD_I_0921) and LuxO (AWOD_I_0920). This finally activates the master regulator LitR (AWOD_I_0419) which regulates the expression of a variety of genes including LuxR.

We were not able to identify any complete QS systems in *M. viscosa*, but genes homologous to *litR* (MVIS_3792) and *luxR* (MVIS_1035) were found. LuxR-family proteins respond to AHLs and can affect the expression of up to 625 target genes in *Vibrio harveyi* alone [33]. If any interspecies communication exists between these two species, the *M. viscosa* LuxR may act as a receptor for AHLs and possible AI-2 produced by *A. wodanis*. The effect of this potential eavesdropping is however not known.

### Winter-ulcer pathogenesis

The winter-ulcer disease aetiology is complex, where “winter-ulcer” refers to infection with *M. viscosa* although additional species of bacteria such as *A. wodanis* and *Tenacibaculum* spp. are recurrently reported isolated. *M. viscosa* is highly virulent in Atlantic salmon [3] and secretes toxic compounds [25]. Skin has been suggested as the initial infection site as ulceration corresponds with direct skin surface colonization [34]. Also *A. wodanis* secretes toxins cytotoxic to fish cell lines, and is able to co-infect (with *M. viscosa*) Atlantic salmon [3]. Likewise is a *Tenacibaculum* sp. shown to produce skin ulcers in scarified areas during challenge and co-infect ulcers already induced by *M. viscosa* [35]. This indicates the presence of multimicrobial communities on fish skin or in ulcers, which are likely to interact with each other. Predisposing Atlantic salmon to *A. wodanis* before *M. viscosa* challenge reduce mortality compared to mono-infection with *M. viscosa* [3]. In this study, *A. wodanis* is also shown to affect *M. viscosa* growth. The encoding bacteriocin locus in *A. wodanis* could in part be the responsible factor that negatively impedes *M. viscosa* growth and thereby indirectly reduce *M. viscosa* virulence*.* Such a reduction in virulence could relate to the chronic development of the winter-ulcer disease observed in field outbreaks compared to the acute phase development in experimental settings where only *M. viscosa* is present. Overall this could imply that the microbial composition present on the surface of a fish is influencing the pathogenesis of skin-mucosal diseases such as winter-ulcer, and that modulating this layer is a potential mechanism to influence the pathogenesis of diseases.

## Conclusions

The two bacteria *M. viscosa* 06/09/139 and *A. wodanis* 06/09/139 were isolated from one Atlantic salmon with winter-ulcer disease and the genomes sequenced. Co-cultivation studies showed that *A. wodanis* is impeding the growth of *M. viscosa*, and that the inhibitorial effect is not contact-dependent. The competition could either come from the fight over nutritional resources, or it could come from that *A. wodanis* is actively producing and secreting bacteriocin-like agents that impede growth across species including both *A. wodanis* and *M. viscosa*. Expression profiles of the transcriptomes supports that the presence of *A. wodanis* affect *M. viscosa*, possibly by altering the global gene expression of *M. viscosa*. Further studies are needed to understand the interplay between *M. viscosa* and *A. wodanis* in causing winter-ulcer disease in fish. The overall findings reported in this study can be useful for future development of alternative bioproducts that could potentially control the survival and health status of farmed fish.

## Methods

### Strains and growth conditions

*A. wodanis* strain 06/09/139 and *M. viscosa* strain 06/09/139, originally isolated from the head kidney of an Atlantic salmon diagnosed with winter-ulcer disease and previously confirmed pathogenic to Atlantic salmon [3] were streaked out onto blood agar (BA) plates (containing blood agar base no. 2, Oxoid CM271, 7 % human whole blood and 2.5 % (w/v) NaCl). Single colonies were selected and expanded for two days at 200 rpm shaking in Luria-Bertani broth (LB) with 1.0 % NaCl (LB1) at 7 °C. In an attempt to mimic their co-existence and growth as observed from field outbreaks, bacteria were grown in tubings *in vitro*, either in mono-culture or in co-culture. Bacteria cultures were diluted to A_600_ ~ 0.015 in LB1 and cultivated in sterile semi-permeable 25 mm diameter regenerated cellulose dialysis tubings with MW cut-off 12–14 000 Da (Sprectra/Por, Los Angeles, CA), immersed in an equal volume of 200 ml LB1. Co-culture growth was also assessed by colony growth distribution. *M. viscosa* and *A. wodanis* grown in LB1 at 7 °C to approximately A_600_ ~ 1.0 were diluted to 10^−1^ suspensions before assorted co-culture mixtures with increasing *M. viscosa* and decreasing *A. wodanis* ratios were made. The co-cultures were spotted in 2 μl aliquots onto BA plates containing 1 % NaCl and incubated at 7 °C. Growth rates in spotted co-culture colonies were examined at initiation and at day 3, 5 and 7 by resuspending entire colonies in LB1 and plating ten-fold serial dilutions onto BA plates.

### Inoculation of dialysis tubings and *in vivo* implantation

Preparation of dialysis tubings was performed after the method of Colquhoun et al. (1998) [36]. Briefly, 20 cm of dialysis tubings (described above) were autoclaved in PBS and added 1 ml (OD 0.6) bacterial suspension cultured in LB1. The model utilized three sets of groups; mono-cultures of either *M. viscosa* or *A. wodanis* in one tube was implanted, the second set where *M. viscosa* and *A. wodanis* were implanted together in separate tubes, and the third set where *M. viscosa* and *A. wodanis* were implanted together in the same tube. Three consecutive knots sealed with silicon followed by ethanol desinfection prevented leakage. The implants were stored in sterile LB1 until implantation (within two hours). Unvaccinated Atlantic salmon obtained from Akvaforsk AS (Sunndalsøra, Sjølseng 12917, Norway) were maintained at the Norwegian Institute for Water Research’s aquaculture station (Solbergstrand, Norway). Fish (~1 kg) were anesthetised (0.0025 % benzocaine solution, Sigma, St. Louis, MO), before executing a small incision (appr. 2 cm long) medially and anteriorly to the pelvic fins. Dialysis tubings were fed into the peritoneal cavity followed by closure of the incision with three sutures (Ethicon, Suturamid 4–0), each passing through all layers of tissue. A solution of benzocaine and fresh seawater was pipetted over the gills periodically during the surgery. All fish survived the surgery and implanted fish were marked by fin clipping. The fish were kept in a 1400 L tank of seawater (salinity 3.1–3.5 %) at 9 °C without feeding. The water quality was monitored throughout the experiment and the oxygen content was maintained stable at 8.2 mg l-1. Fish were euthanized on day 4 with an overdose of benzocaine and a blow to the head. Bacterial cultures from the recovered implants were briefly centrifuged (14 000 x *g*, 2 min, 4 °C) before snap frozen in liquid nitrogen (within 3 min after removal from the fish).

### Whole genome sequencing

The two *M. viscosa* 06/09/139 and *A. wodanis* 06/09/139 genomes have been completely sequenced using a combination of 454, Illumina and Pacific Bioscience sequencing technology. Total bacterial DNA was isolated using proteinase K treatment followed by chloroform:phenol extraction. Initially, the 454 data was *de novo* assembled with Newbler [37], and further used as a scaffold for the assembly of the Illumina data using IMAGE [38]. The scaffolds were ordered with regards to the complete genomes of closely related species using ABACAS [39], and remaining gaps closed by sequencing positive PCR products bridging contigs in the assembly.

### Functional annotation and pathway analysis

Both genomes have been functionally annotated as described previously [40] using Artemis software [41], and are available from EMBL/GenBank/DDBJ with the accession numbers PRJEB6964 and PRJEB6963. Additionally, Priam [42] and KEGG Mapper [43] was used to predict enzymes and visualize metabolic pathways, respectively.

### RNA preparation and cDNA synthesis

RNA was isolated using the MasterPure™ Complete DNA and RNA Purification Kit (EPICENTER, Cat. Nos. MC85200 and MC89010) according to manufacturer’s instructions. RNA samples were quantified by ND-1000 Spectrophotometer (NanoDrop®Technology, DE, USA) and its integrity controlled by agarose gel electrophoresis in all successive steps. rDNase I included in the DNA-Free™ Kit (Ambion) was used to remove excess genomic DNA according to manufacturer’s instructions. Samples were confirmed DNA-free with a 16S rDNA primer set to a level below PCR detection and inactive for DNase by digestion analysis. Twenty five μg of each total RNA extract was reversely transcribed using random hexamers, 40U RNaseOUT™ and 400U Superscript III RT (Invitrogen) at 42 °C for two hours. Denaturation occurred at 70 °C for 20 min. Transcription products were purified using Illustra AutoSeq G-50 columns (GE Healthcare) and by Trizol (GibcoBRL) extraction. cDNA synthesis was confirmed by PCR.

### Library construction and sequencing

The single-stranded cDNA libraries were constructed as described in [44]. Gel electrophoresis was used to select for DNA constructs approximately 200 bp in size, and amplified by PCR. The libraries were loaded onto two separate lanes of an Illumina GA flow cell and sequencing reactions performed according to the manufacturer’s recommended protocol.

### Read mapping and visualisation

The cDNA sequence reads were mapped uniquely to the genomes of *M. viscosa* 06/09/139 and *A. wodanis* 06/09/139, and quantification of transcript abundance and calculation of differential gene expression were performed using Rockhopper [45]. Gene expression levels were quantified as the number of sequence reads mapping to the sequence of the gene divided by the length of the gene and the upper quartile of gene expression. Rockhopper use the Negative Binomial distribution as a model to compute p-values, and we regarded p > 0.05 as the probability of observing a transcript’s expression levels in different conditions by chance.

### PCR screening and bacteriocin assay

Total DNA preparation from all bacterial isolates, PCR amplification and amplicon visualization (Additional file 5) of the putative plasmid bacteriocin operon was performed identical to [3].

*A. wodanis* isolates, listed in Additional file 5, were screened for production of bacteriocin. Isolates were grown in 3 ml LB 3.5 % NaCl at 10 °C with shaking. Cultures (100 μl) were diluted and further expanded for two days in 3 ml LB1 at 10 °C with shaking. Cultures were adjusted to OD_600_ ~ 0.4 and 100 μl were inoculated in 3 ml fresh LB1 at 10 °C and grown to approximately A_600_ ~ 0.25 to 0.35. A culture loop (1 μl) of one of the bacterial suspensions was then streaked in a single straight line in the centre of BA plates (containing a total of 1.0 % NaCl and supplemented with 5 % bovine blood). A loopfull from each culture was streaked perpendicular to this traversing the original streaked suspension. This method was followed for all isolates. Plates were incubated at 8 °C for 5 days before being examined for growth inhibition patterns. To confirm antimicrobial activity production 2 μl of the bacterial suspensions were spotted onto Luria agar containing 1.0 % NaCl (LA1) incubated at 8 °C for 3 days. Plates and colonies were then overlaid with a new layer of LA1 and 10^3^ serial diluted bacterial suspensions (A_600_ ~ 0.4) were plated on top. Plates were incubated 5 days at 8 °C. Colonies surrounded by clear zones in the on-top bacterial lawn indicated bacteriocin activity.

### Ethical approval

The study “*In vivo* communication between winter ulcer bacteria *Moritella viscosa* and *Vibrio wodanis*”, Id. Nr 2041 was approved by the Norwegian National Animal Research Authority in August 26. 2009.

## Availability of supporting data

The annotated genomes have been deposited to the European Nucleotide Archive ENA (http://www.ebi.ac.uk/ena/) with the accession numbers PRJEB6964 and PRJEB6963.

The Illumina reads from the RNA sequencing have been deposited to the Sequence Read Archive repository at ENA and they are available under study accession number ERP006698, and the meta data to ArrayExpress under accession number E-MTAB-2851.

## Additional files

Additional file 1:
**Schematic circular diagrams of the replicons of A)** ***M. viscosa*** **06/09/139 and B)** ***A. wodanis*** **06/09/139.** Appropriate categories are shown as pairs of concentric circles representing both coding strands. Key to the chromosomal circular diagrams (outside to inside): scale (in Mb), annotated CDS, pseudogenes (brown), non-coding and structural RNA; rRNA (blue), tRNA (green) and sRNA (pink), % G + C content, G + C deviation (>0 % olive, <0 % purple). Colour coding for CDSs (according to predicted function): dark blue, pathogenicity/adaptation; black, energy metabolism; red, information transfer; dark green, surface associated; cyan, degradation of large molecules; magenta, degradation of small molecules; yellow, central/intermediary metabolism; pale green, unknown; pale blue, regulators; orange, conserved hypothetical; brown, pseudogenes; pink, phage + IS elements; grey, miscellaneous. Additional file 2:
**Growth curves of** ***A. wodanis*** **06/09/139 and** ***M. viscosa*** **06/09/139 at 7 °C.** Colonies from blood agar plates were expanded over-night in LB with 3.5 % NaCl at 7 °C. The cultures were used to inoculate fresh LB with either 1.0 % or 3.5 % NaCl to a concentration of A_600_ ~ 0.02. Cultures were expanded to exponential phase (*A. wodanis* A_600_ ~ 0.8, *M. viscosa* A_600_ ~ 0.4) and further used to inoculate fresh LB with either 1.0 % or 3.5 % NaCl to a concentration of A_600_ ~ 0.02. Density measurements were obtained regularly for triplicates of each condition and is presented as A_600_ average ± standard deviation. Additional file 3:
***M. viscosa*** **and** ***A. wodanis*** **genes that are more than 2-fold up- or down regulated (p-value < 0.05) in co-infection versus single infection.**
Additional file 4:
**Comprison of the siderophore biosynthesis, secretion and uptake locus in** ***A. wodanis*** **06/09/139 and the vibrioferrin locus of** ***V. parahaemolyticus*** **WP1.** Similarities of the translated products are shown in grey. Additional file 5:
***A. wodanis*** **isolates screened for the presence of bacteriocin locus genes.**
Additional file 6:
**Antimicrobial activity assays by cross streaking isolates on blood agar plates and by observing inhibition zones on the soft agar overlay.**

## Abbreviations

QS: Quorum sensing
AHL: N-acyl homoserine lactone
BA: Blood agar
cfu: colony forming unit
