# Supplementary figures and images for: Co-cultivation and transcriptome sequencing of two co-existing fish pathogens Moritella viscosa and Aliivibrio wodanis
Source: BMC Genomics. 2015 Jun 10;16(1):447. doi: 10.1186/s12864-015-1669-z (PMC4462113; doi:10.1186/s12864-015-1669-z)

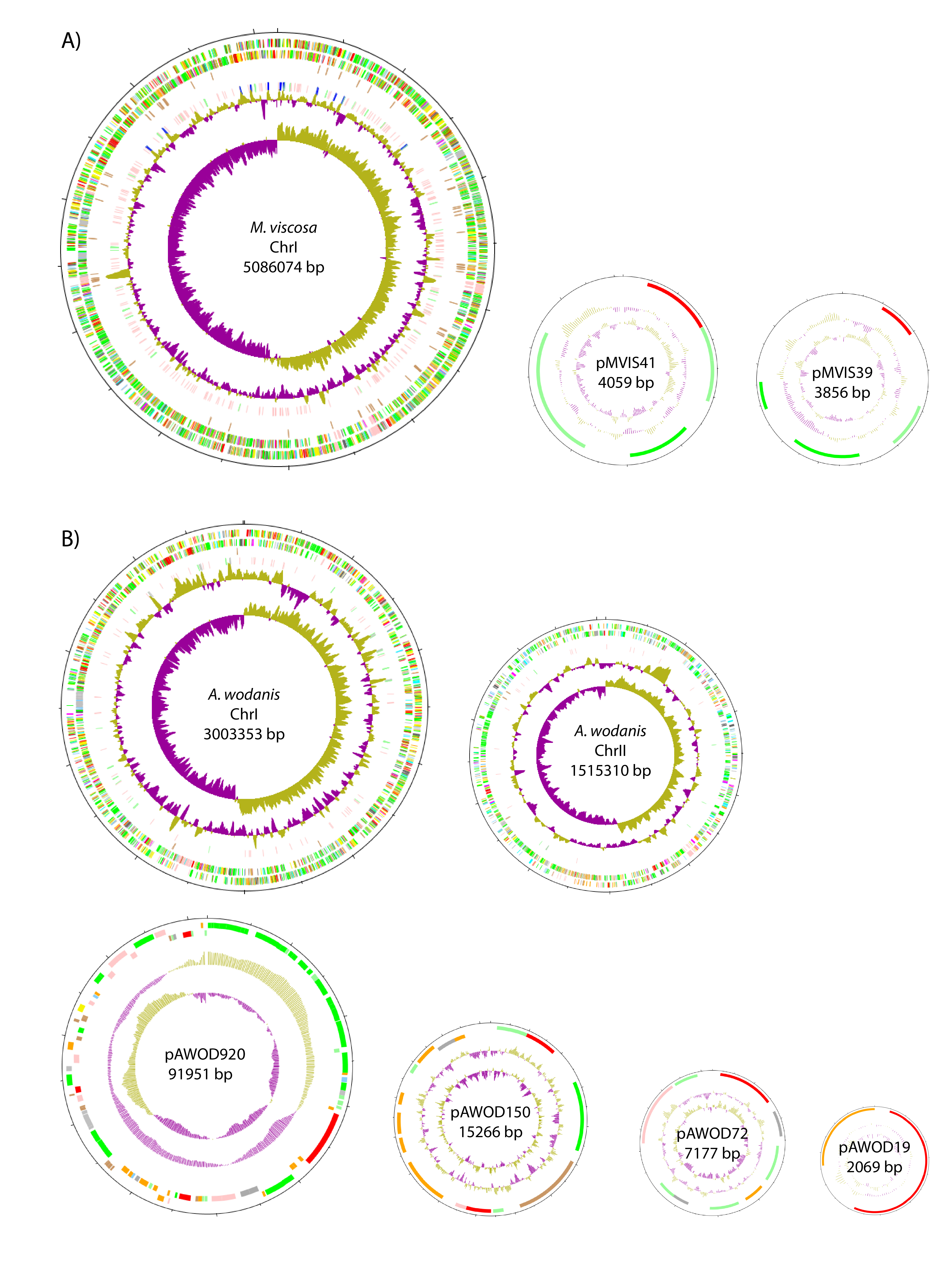

Supplement: Additional file 1: — Schematic circular diagrams of the replicons of A) M. viscosa 06/09/139 and B) A. wodanis 06/09/139. Appropriate categories are shown as pairs of concentric circles representing both coding strands. Key to the chromosomal circular diagrams (outside to inside): scale (in Mb), annotated CDS, pseudogenes (brown), non-coding and structural RNA; rRNA (blue), tRNA (green) and sRNA (pink), % G + C content, G + C deviation (>0 % olive, <0 % purple). Colour coding for CDSs (according to predicted function): dark blue, pathogenicity/adaptation; black, energy metabolism; red, information transfer; dark green, surface associated; cyan, degradation of large molecules; magenta, degradation of small molecules; yellow, central/intermediary metabolism; pale green, unknown; pale blue, regulators; orange, conserved hypothetical; brown, pseudogenes; pink, phage + IS elements; grey, miscellaneous. [file 12864_2015_1669_MOESM1_ESM.jpeg]

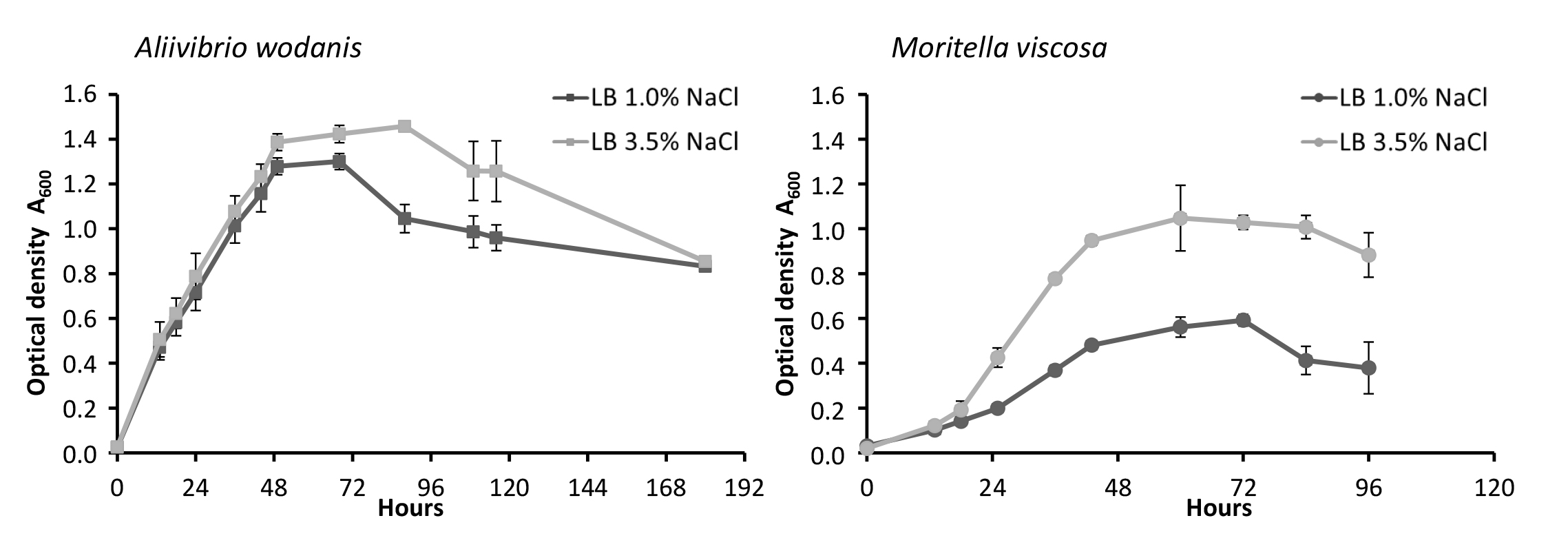

Supplement: Additional file 2: — Growth curves of A. wodanis 06/09/139 and M. viscosa 06/09/139 at 7 °C. Colonies from blood agar plates were expanded over-night in LB with 3.5 % NaCl at 7 °C. The cultures were used to inoculate fresh LB with either 1.0 % or 3.5 % NaCl to a concentration of A600 ~ 0.02. Cultures were expanded to exponential phase (A. wodanis A600 ~ 0.8, M. viscosa A600 ~ 0.4) and further used to inoculate fresh LB with either 1.0 % or 3.5 % NaCl to a concentration of A600 ~ 0.02. Density measurements were obtained regularly for triplicates of each condition and is presented as A600 average ± standard deviation. [file 12864_2015_1669_MOESM2_ESM.jpeg]

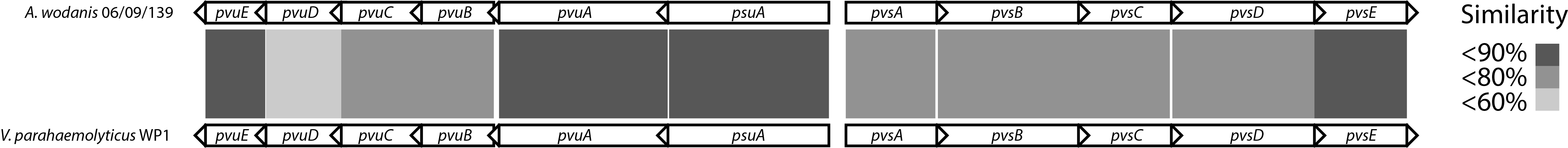

Supplement: Additional file 4: — Comprison of the siderophore biosynthesis, secretion and uptake locus in A. wodanis 06/09/139 and the vibrioferrin locus of V. parahaemolyticus WP1. Similarities of the translated products are shown in grey. [file 12864_2015_1669_MOESM4_ESM.jpeg]
